# Selective mode of action of plumbagin through BRCA1 deficient breast cancer stem cells

**DOI:** 10.1186/s12885-016-2372-4

**Published:** 2016-05-26

**Authors:** Veena Somasundaram, Sreelatha K Hemalatha, Krishnendu Pal, Sutapa Sinha, Asha S. Nair, Debabrata Mukhopadhyay, Priya Srinivas

**Affiliations:** Cancer Research Program, Rajiv Gandhi Centre for Biotechnology, Thiruvananthapuram, Kerala 695014 India; Department of Biochemistry and Molecular Biology, Mayo Clinic College of Medicine, Rochester, MN 55905 USA; Department of Hematology, Mayo Clinic College of Medicine, Rochester, MN 55905 USA; Present address: Center for Cancer Research, National Cancer Institute, Building 567, Room 254, Frederick, MD 21702-1201 USA

**Keywords:** BRCA1, Cancer stem cells, Plumbagin, ALDH1

## Abstract

**Background:**

Studies over the past decade and half have identified cancer stem cells (CSCs) to be responsible for tumorigenesis, invasion, sustenance of metastatic disease, radio- and chemo-resistance and tumor relapse. Recent reports have described the plasticity of breast CSCs (BCSCs) to shift between the epithelial and mesenchymal phenotypes via Epithelial-Mesenchymal Transition (EMT) and Mesenchymal-Epithelial Transition (MET) states as the reason for their invasive capabilities. Additionally, BRCA1 has been found to be a mammary stem cell fate determinant. However, it is not clear what would be the best marker that can be used for identifying CSCs in BRCA1 mutated cancers. Also, anticancer agents that can reduce CSC population in a BRCA1 defective condition have not been addressed so far.

**Methods:**

Putative BCSCs were identified based on Hoechst exclusion, CD44^+^/24^–/low^ expression and Aldehyde Dehydrogenase 1 (ALDH1) positivity using flow cytometry. The ‘stemness’ of the isolated ALDH1+ cells were analysed by immunofluorescence, western blotting for stem cell and EMT markers as well as in vitro mammosphere assays. Induction of Reactive Oxygen Species (ROS) by Plumbagin (PB) in BCSCs was assayed by Dichloro-dihydro-fluorescein diacetate (DCF-DA) staining. Ovarian cancer xenografts treated with PB were subjected to immunohistochemical analysis to study the ability of PB to target CSCs.

**Results:**

We have confirmed that ALDH1 positivity is the best marker for the identification of BCSCs in BRCA1-defective breast cancer cell lines when compared to the CD marker profile and Side Population (SP) analysis. BRCA1 status was observed to be a determinant of the abundance of epithelial-like (ALDH1+) or mesenchymal-like (CD44^+^/24^–/low^) BCSCs, and the reconstitution of a full length, wild type BRCA1 in HCC1937 breast cancer cells possessing a mutated BRCA1, transforms them from ‘stem-like’ to more ‘mesenchymal’. For the first time we have shown that Plumbagin (PB), a naturally occurring naphthoquinone which is predominantly a ROS inducer, could reduce BCSCs specifically in BRCA1-defective, basal-like cancer cells.

**Conclusions:**

The best marker for identifying BCSCs in BRCA1 defective condition could be ALDH1 and that BRCA1 mutated BCSCs would be mostly ‘stem like’ than ‘mesenchymal’. Also ROS inducers like PB could reduce BCSCs in BRCA1 defective cancers.

**Electronic supplementary material:**

The online version of this article (doi:10.1186/s12885-016-2372-4) contains supplementary material, which is available to authorized users.

## Background

In 1858, Rudolf Virchow proposed the embryonal rest theory of cancer [1]. This was the first ever reference to the possibility that a stem cell that stayed dormant during embryonic development and for years later could eventually become a cancer initiator. Julius Conheim corroborated this in striated muscle sarcoma of the kidneys [2]. After ‘resting’ for almost a century and half since its first mention, the embryonal rest theory was revived as the CSC hypothesis and then began the hunt for biomarkers to identify CSCs. Once the existence of a therapy resistant population within a tumor that could possibly be the origin of tumorigenesis as well the reason for relapse was scientifically shown in leukemia [3], the Cancer Stem Cell hypothesis has been rapidly gaining support from various quarters. The existence of CSCs in breast cancer was first brought to light when it was found that the CD44^+^CD24^−/low^Lin^−^ cells from breast cancer patients were able to generate tumors more efficiently than CD44^+^CD24^+^Lin^−^ cells when implanted into the mammary fat pads of NOD/SCID mice [4]. Also, these cells exhibited the typical stem-like characteristics of self-renewal (surviving serial passage) and differentiation (reproducing the tumor heterogeneity as in the primary tumor). Subsequently, a number of markers, including ALDH1 were discovered for BCSCs with special reference to the different molecular sub-types with many of the markers being common to normal mammary stem cells as well as BCSCs [5–14]. However, most biomarkers till date have not stood the test of time. This, in addition to the difficulties of accurate xenograft studies have prevented the CSC hypothesis from evolving into a theory.

The best possible method would be to restrict a study to a single subtype of cancer and try to find markers specific to CSCs in the said cancer type as the dearth of currently known distinct, distinguishing features for identification of CSCs means that there is a long way to go in the identification (if at all) of specific CSC marker/s for every cancer type.

BRCA1-defective tumors represent a class of early onset, high grade, poor prognosis, often triple negative breast cancers (TNBCs) with no specific or selective treatment regimen [15]. BRCA1 is a transcriptional regulator and plays a cardinal role in Homologous Recombination Repair of damaged DNA [16–18]. Later, it was found to be important in embryonic development and was subsequently reported to be a determinant of the mammary stem cell fate [19, 20]. However, the characteristics of BCSCs from BRCA1-defective cancers are largely unexplored. Earlier studies from our group have revealed the ability of PB (5-hydroxy-2-methyl-1, 4-naphthoquinone) to selectively target BRCA1-defective ovarian cancer cells [21–24] as well as BRCA1-knockout breast cancer xenografts (unpublished data). Even though PB acts via multiple pathways to induce cytotoxicity in cancer cells, the major mode of action is the generation of ROS and subsequent DNA damage which could be especially deleterious to BRCA1-defective cancer cells that lack the homologous recombination repair machinery [21–28]. Therefore, in this study we analyzed the ability of PB to target BCSCs from BRCA1-defective cancers.

Here we report that ALDH1 positivity could be one of the best markers for the identification of BCSCs from basal-like, BRCA1-defective breast cancer cell lines based on the isolation of putative CSCs followed by their in vitro mammosphere culture. We have also shown for the first time that PB can target CSCs wherein the BCSCs derived from the cells possessing a reconstituted BRCA1, show an enrichment of ALDH1+ cells upon PB treatment. The BRCA1-defective HCC1937 shows a robust down regulation of ALDH1 positivity after treatment with sub-cytotoxic doses of PB. On the other hand, the standard drug carboplatin (CP) increased ALDH1+ cells in both the cell lines, which might be one of the reasons for relapse after CP treatment.

## Methods

### Cell culture experiments

Human breast epithelial adenocarcinoma cell lines (MCF7, MDA-MB-231, MDA-MB-436), and a human ovarian adenocarcinoma cell line (OVCAR-5) were purchased from American Type Culture Collection (ATCC, Manassas, VA, USA). Human breast ductal carcinoma cell line; HCC1937 and HCC1937 reconstituted with wild type BRCA1 (HCC1937/wt BRCA1) were kind gifts from Dr. Grant Mc Arthur, Peter MacCallum Cancer Centre, VIC, Australia. All the cell lines were maintained in culture medium containing 10 % Fetal Bovine Serum (FBS) (PAN-Biotech GmBH, Aidenbach, Germany), 0.1 g/L Streptomycin (Sigma Aldrich, St. Louis, MO, USA) and 100U/L Penicillin in a humidified incubator at 37 °C in 5 % (v/v) CO_2_. MCF7, MDA-MB-231 and OVCAR-5 were maintained in Dulbecco’s Modified Eagles’ Medium (Gibco, Carlsbad, CA, USA), MDA-MB-436 in RPMI 1640 (PAN-Biotech GmBH, Aidenbach, Germany) and HCC1937 and HCC1937/wt BRCA1 in RPMI 1640 with insulin (5 μg/ml). Passage numbers post thawing of the cell lines were noted and experiments were carried out within a maximal passage number (<30).

### Cell viability assay

MTT assay to assess the effects of 48 h treatment with PB and CP on HCC1937 and HCC1937/wt BRCA1 was performed as described elsewhere [23]. 3–(4,5-Dimethyl thiazol-2-yl) 2,5-diphenyl tetrazolium bromide (MTT) was purchased from USB, Cleveland, OH, USA, PB and CP were from Sigma Aldrich, St. Louis, MO, USA. CP solutions were prepared in phosphate buffered saline (PBS) immediately before the experiment. PB was dissolved in Dimethyl Sulfoxide (DMSO) and the vehicle control (DMSO) did not show any effect on cell viability. All results are expressed as the percentage cell viability over control ± S.D. of quadruplicate determinations from three independent experiments.

### Side population analysis

Cells were stained with 5 μg/ml of Hoechst 33,342 (Sigma Aldrich, St. Louis, MO, USA) for 90 min in a 37 °C water bath. Cells stained with 5 μg/ml Hoechst in the presence of 50 μM Verapamil Hydrochloride (calcium channel blocker that prevents Hoechst efflux from cells) (Sigma Aldrich, St. Louis, MO, USA) was used as negative control. The cells were then stained with propidium iodide (PI) (2 μg/10^6^ cells) for dead cell exclusion and sorted using BD FACSAriaII flow cytometer (Becton Dickinson, Franklin Lakes, NJ, USA). Hoechst 33,342 stained cells were analyzed using 350 nm excitation with blue (635 nm) and red (488 nm) emission. BD FACSDiva Software was used for analysis.

### Surface marker profiling

Cells were stained with Mouse anti-human CD44-APC (C26) and CD24-FITC (ML5) antibodies (BD Biosciences (San Jose, CA., USA)) for 30 min on ice. Cells stained with CD44-APC and CD24-FITC separately (single color) were taken as the controls. The 633 nm and 488 nm lasers were used for excitation and 530/30 nm BP and 660/20 BP detectors were used for CD24-FITC and CD44-APC respectively. The CD44^+^/24^–/low^ (putative stem cell) population was identified and analyzed using flow cytometry as mentioned above.

### ALDEFLUOR assay

The ALDEFLUOR assay (The ALDEFLUOR Assay kit, Stem Cell Technologies (Durham, NC, USA)) was used to identify and isolate the ALDH+ cells from the various cell lines. Briefly, cells in growing conditions or treated with PB or CP for 48 h were suspended in ALDEFLUOR assay buffer containing ALDH substrate (BODIPY-aminoacetaldehyde or BAAA, 1 μmol/l per 1 × 10^6^ cells) and incubated for 40 min at 37 °C in a water bath to assess the ALDH enzymatic activity. As negative control, an aliquot was treated with 50 mmol/l diethylaminobenzaldehyde (DEAB), a specific ALDH inhibitor for each sample of cells. The ALDH1 positive (ALDH1+) and ALDH1 negative (ALDH1-) populations were sorted out by flow cytometry using the 488 nm laser and the 530/30 nm BP detector as mentioned above.

### In vitro propagation of BCSCs: mammosphere culture

Cells were seeded for primary mammosphere formation at 2000 cells/ml in ultralow attachment plates (Corning Inc., Corning, NY, USA) in MEBM (Mammary Epithelial Basal Medium Bullet kit from Lonza (Basel, Switzerland)) containing insulin (5 μg/ml) hydrocortisone (1 μg/ml), EGF (10 ng/ml), bFGF (20 ng/ml) (BD Biosciences, San Jose, CA), 2 % B27 serum free supplement (Life Technologies, Carlsbad, CA, USA) and heparin (4 μg/ml) (Sigma Aldrich, St. Louis, MO, USA). Medium was supplemented every 4 days. Mammospheres (>50 μm) were counted and photographed on day 7. The cells were treated with sub-cytotoxic concentrations of PB (single dose) and cultured for 7 days to study the sphere forming efficiency and ABCG2 expression. During the treatment, growth medium without PB was supplemnted every 4 days.

### 3 D culture of spheroids

*In order to recapitulate the mammosphere data in a system that involves the extracellular matrix components as would be seen in vivo*, cells were seeded at 10,000 cells/ml in RPMI with insulin (5 μg/ml /ml), hydrocortisone (1 μg/ml), EGF (10 ng/ml) and 5 % Growth Factor Reduced Matrigel (GFRM) to 8-well chamber slides (BD Falcon, San Jose, CA, USA) pre-coated with 50 μl GFRM (BD Biosciences, San Jose, CA, USA). 0.5 μM PB was added to the cells. Cells were viewed on Day 5, Day 7, Day 10 and Day 15 using the Olympus IX71 microscope and photographed.

### Immunofluorescence analysis

Day 7 mammospheres were washed with Phosphate Buffered Saline (PBS) and fixed in 4 % paraformaldehyde. Following permeabilization with 0.25 % Triton × 100, and blocking with 1 % Bovine Serum Albumin (BSA), spheres were incubated overnight at 4 °C in the primary antibody. After PBS wash and incubation with fluorochrome tagged secondary antibody for 2 h, spheres were mounted in Prolong Gold Antifade with DAPI (Life Technologies, Carlsbad, CA, USA) and viewed using the Olympus IX71 microscope. Immunofluorescence analysis was performed with Rabbit anti-human Snail + Slug, rabbit anti-human β catenin (E247), rabbit anti-human α-SMA (Smooth Muscle Actin), and goat anti-human Oct 4 purchased from Abcam (Cambridge, MA, USA), goat anti-human Vimentin (C20), rabbit anti-human BRCA1, mouse anti-human ABCG2 (6D17) from Santa Cruz Biotechnology (Santa Cruz, CA, USA).

### DCF-DA staining

ROS production in mammospheres was assessed from the levels of bright green colored 2′,7′-dichlorofluorescein (DCF), produced by the oxidation of DCF-DA (2′,7′-Dichlorodihydrofluorescein diacetate) dye by ROS induced in mammospheres after a 10 min treatment with 1 μM PB followed by exposure to 500 nM DCF-DA (Sigma Aldrich, St. Louis, MO, USA) (stock dissolved in Dimethyl formamide and further diluted in PBS) for 20 min in dark at 37 °C. The green fluorescence was detected by the Leica DMI 6000B microscope at 495/529 nm.

### Comet assay

A single gel electrophoresis method for comet assay with modifications as described by Olive et al., 2006 was done. Briefly, cells treated with PB for 4 h were lysed using neutral lysis buffer overnight at 37 °C and seeded on to low melting agarose gel in a frosted slide. Then electrophoresis was done in Tris Borate EDTA buffer for 30 min at 50 V. The slides were then stained with propidium iodide and observed under an Olympus 1X71 fluorescent microscope for comets. The comets formed were scored using Casplab software.

### Western blotting

Cell lysates were analyzed for expression of ABCG2, β-catenin and β-actin. Whole cell lysates were prepared in RIPA buffer supplemented with protease inhibitor cocktail. Supernatant was collected by centrifugation at 13,000 rpm for 25 min at 4 °C. Samples were then subjected to sodium dodecyl sulphate polyacrylamide gel electrophoresis (SDS-PAGE), transferred to nitrocellulose membranes and immunoblotted. Protein bands were observed by Enhanced Chemiluminescence detection (Amersham, Piscataway, NJ, USA) of the specifically bound antibody. The quantitation was done by densitometric analysis by “Quantity One” software.

### Xenograft experiments

Six-week old female Severe Combined Immunodeficient (SCID) mice with tumors left untreated for 28 days were randomized into two groups (ten animals in a group). Group 1 was treated with 25 % polyethylene glycol (PEG) alone. Group 2 was treated with PB in 25 % PEG at doses of 1 mg/kg/day intraperitoneally. Tumors were measured twice a week, and primary tumor volumes were calculated using the formula V = 1/2a x b^2^, where ‘a’ is the longest tumor axis, and ‘b’ is the shortest tumor axis.

### Immunohistochemical analysis (IHC)

After 3 weeks of treatment, all OVCAR-5 tumor-bearing mice were sacrificed by asphyxiation with CO_2_; tumors were removed, measured, and prepared for IHC. Tumors were removed and fixed in neutral buffered 10 % formalin at room temperature for 24 h. Subsequently, the samples were embedded in paraffin and sections were taken. Sections were deparaffinized and then subjected to Oct 4 (Cell Signaling Technology, Beverly, MA), Vimentin (Clone V9, Chemicon International, Temcula, CA) and N-cadherin (Clone 13A9, Santa Cruz Biotechnology, Santa Cruz, CA) according to the manufacturer’s instructions (DAB 150, (Millipore, Billerica, MA). The secondary anti-mouse antibody was used before adding chromogen substrate. Stable diaminobenzidine was used as a chromogen substrate, and the sections were counterstained with a hematoxylin solution. Photographs of the entire cross-section were digitized using an Olympus camera (DP70).

### Statistical analysis

The independent-sample paired two tailed student t-test was used to test the probability of significant differences between different experimental groups. FACS results were expressed as mean ± S.D from at least three independent experiments. Statistical significance was defined as (*) *p* ≤ 0.05 and (**) *p* ≤ 0.005. Error bars were given on the basis of calculated S.D values. All experiments were repeated at least thrice.

## Results

### Intrinsic tumor subtype and BRCA1 status can determine the percentage of the BCSCs within cancer cells

**Fig. 1:**
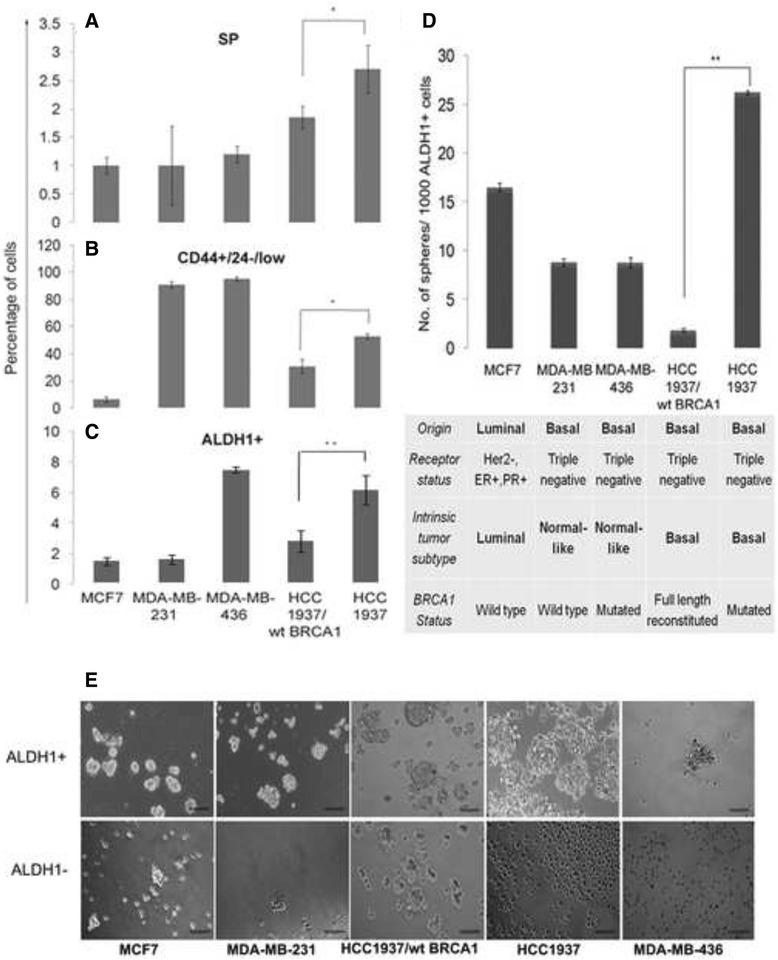
ALDH1+ cells and not ALDH1- cells from BRCA1-defective breast cancer cell lines can form spheres. Graph showing the percentages of SP (**a**), CD44^+^/24^–/low^ (**b**) and ALDH1+ (**c**) cells among the various breast cancer cell lines employed in this study. By all these three methods of identification, the CSC population was significantly higher in BRCA1-defective HCC1937 when compared to HCC1937/wt BRCA1. **d** and (**e**) A comparison of the in vitro sphere forming ability of ALDH1+ and ALDH1- cells from various cell lines indicating the inability of ALDH1- cells from BRCA1-defective cell lines to form spheres thus supporting that the ALDH1+ population from these cell lines entirely represents the BCSCs. Scale = 100 μm

A comparison of the variations in the percentages of SP, CD44^+^/24^–/low^ and ALDH1+ cells among breast cancer cell lines of different origins with varied receptor status has not been reported. Hence, we studied the effects of varied receptor status and possible effects of BRCA1 gene expression on these subcellular populations. The cells showing low staining at both 635 nm and 488 nm were identified as the Hoechst excluding side population (SP) cells. Hoechst excluding SP cells constituted less than 2 % of the total breast cancer cell population in four out of the five cell lines analyzed. The percentage of SP in HCC1937 (2.7 ± 0.42 %) was about 1.5 times higher than that in HCC1937/wt BRCA1 cells (1.85 ± 0.21 %) (*p* < 0.05) (Fig. 1a). The putative BCSCs were also identified in all the five cell lines and isolated based on the CD44^+^/24^–/low^ marker profile as described previously [6, 14]. Studies have suggested that the percentage of CD44^+^/24^–/low^ putative CSCs present would vary between cancer cell lines and this percentage is determined by two main factors: the origin of the cell line and the receptor status [9]. Among the normal-like tumor forming cell lines, the BRCA1-mutated MDA-MB-436 harbored more CD44^+^/24^–/low^ cells (95 ± 1.4 %) than MDA-MB-231 (90.67 ± 1.75 %) (Fig. 1b). Similarly, in basal-like tumor forming cell lines, the CD44^+^/24^–/low^ cells were enriched in BRCA1-defective HCC1937 (52.7 ± 1.84 %) when compared to HCC1937/wt BRCA1 (30.6 ± 5.0 %) that possesses a wild type BRCA1. Thus, the CD44^+^/24^–/low^ expression profile paralleled a BRCA1 defect though the general predominance exhibited tumor sub-type specificity with normal-like tumor forming cells (MDA-MB-436 and MDA-MB-231) possessing more CD44^+^/24^–/low^ cells than the basal-like tumor inducing cell lines (HCC1937 and HCC1937/wt BRCA1).

A tumor sub-type independent increase in numbers of ALDH1+ cells was observed in BRCA1-mutated cell lines MDA-MB-436 (8.4 ± 0.2 %) and HCC1937 (6.9 ± 0.5 %) when compared to cells possessing a wild type BRCA1 (Fig. 1c). ALDH1+ cells from HCC1937 showed maximum sphere forming capability followed by MCF7. MDA-MB-231 and MDA-MB-436 formed comparable number of spheres after 7 days in culture while HCC1937/wt BRCA1 cells showed the least capability for in vitro mammosphere formation (Fig. 1d). The ALDH1 negative (ALDH1-) cells from HCC1937 and MDA-MB-436 were unable to form spheres in culture when compared to ADH1- cells isolated from MCF7, MDA-MB-231 and HCC1937/wt BRCA1 cell lines which formed a few spheres in non-adherent culture (. 1E). Hence, ALDH1, as a BCSC marker showed a specific association with BRCA1-defective breast cancers and BRCA1 has a cardinal effect on BCSC numbers and properties.

The inclusion of five cell lines with largely varied genotypes made it difficult to draw corroborating conclusions from confirmatory experiments. The two isogenic cell lines HCC1937 and HCC1937/wt BRCA1 that differed exclusively in the expression and functionality of BRCA1, showed significant differences in sphere forming capability (*p* < 0.005), a hallmark of CSCs. Hence, rest of the study is centered on these two TNBC cell lines.

### BRCA1 can influence the EMT traits and proliferative potential of BCSCs

**Fig. 2:**
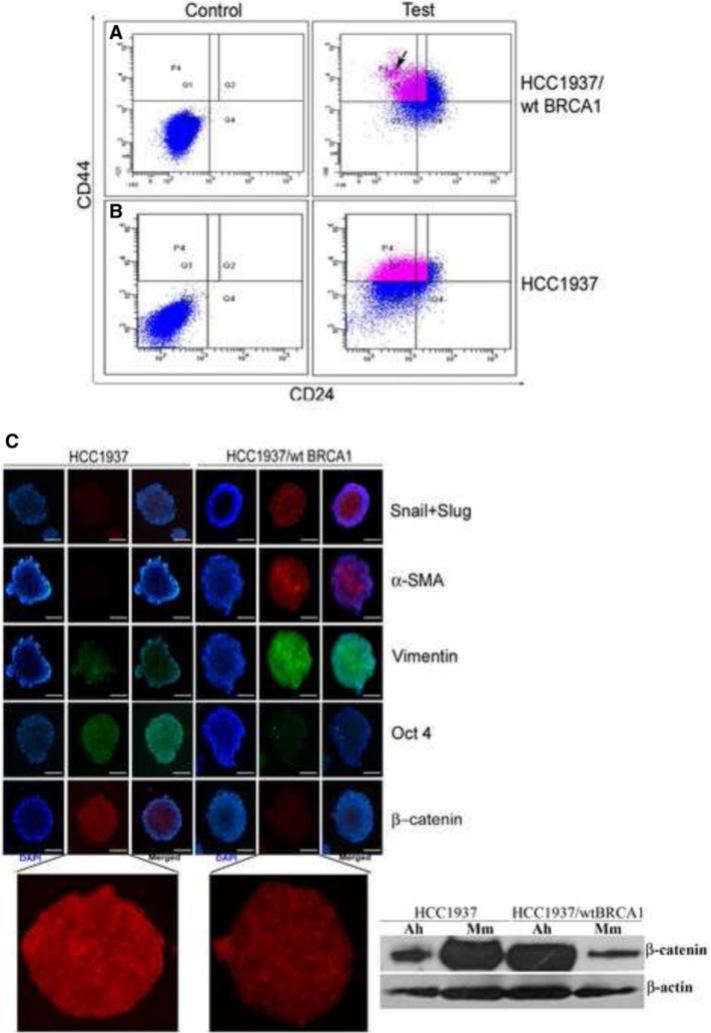
BRCA1 determines the frequency of stem cells or EMT marker expressing cells within cell lines. **a** The distinct CD44^high^/24^–^population (represented by the black arrow) possessing mesenchymal properties is observed within the CD44^+^/24^–/low^ cells (P4) in HCC1937/wt BRCA1. **b** Such a population is not discernable within the HCC1937 cell line. **c** Marker profiling of mammospheres shows better expression of EMT markers (Snail, Slug, Vimentin) and mesenchymal marker (α-SMA) by HCC1937/wt BRCA1 cells. Expression of stem cell markers (Nuclear Oct 4 and membrane bound β-catenin) was more prominent in the HCC1937 cell line as observed by immunofluorescence as well as western blotting. Scale = 50 μm. “Ah” and “Mm” in western blot represents Adherent and Mammospheres respectively

To further assess how BRCA1 influences cancer stemness, we compared and contrasted between the EMT and stem cell marker expression patterns in HCC1937 and HCC1937/wt BRCA1. HCC1937/wt BRCA1 cells showed the presence of a distinct CD44^high^/24^–^population (3.07 ± 1.12 %) within its CD44^+^/24^–/low^ (30.6 ± 5.0 %) putative cancer stem cell population (Fig. 2a, Additional file 1: Figure S1). This population was not observed in HCC1937 cells (Fig. 2b). Studies have attributed the presence of such a population to a more mesenchymal phenotype that parallels EMT traits in cell lines [29, 30]. A comparison of the expression of the EMT markers [Snail, Slug, Vimentin, α-SMA (mesenchymal marker)] and the stem cell markers (Oct 4 and β-catenin) indicated that mammospheres of HCC1937/wt BRCA1 exhibited a higher expression of the EMT markers while HCC1937 showed a higher expression of the stem cell markers (Fig. 2c). HCC1937 mammospheres displayed a membrane localization of β-catenin indicative of retention on cell membrane possibly prior to nuclear localization following activation of Wnt signaling, while HCC1937/wt BRCA1 mammospheres showed slight membrane as well as diffuse cytosolic expression. The cytoplasmic expression of β-catenin is indicative of the absence of Wnt signaling and hence induction of proteosomal degradation of the protein [31]. Additionally, β-catenin expression levels were also much higher in mammospheres of HCC1937 than HCC1937/wt BRCA1, while parental, adherent cells exhibited an opposite trend (Fig. 2c). These observations were corroborated by the microarray analysis of HCC1937 and HCC1937/wt BRCA1 mammospheres (Additional file 2: Table S1).

### Plumbagin shows selective sensitivity for BRCA1 defective breast cancer cells

**Fig. 3:**
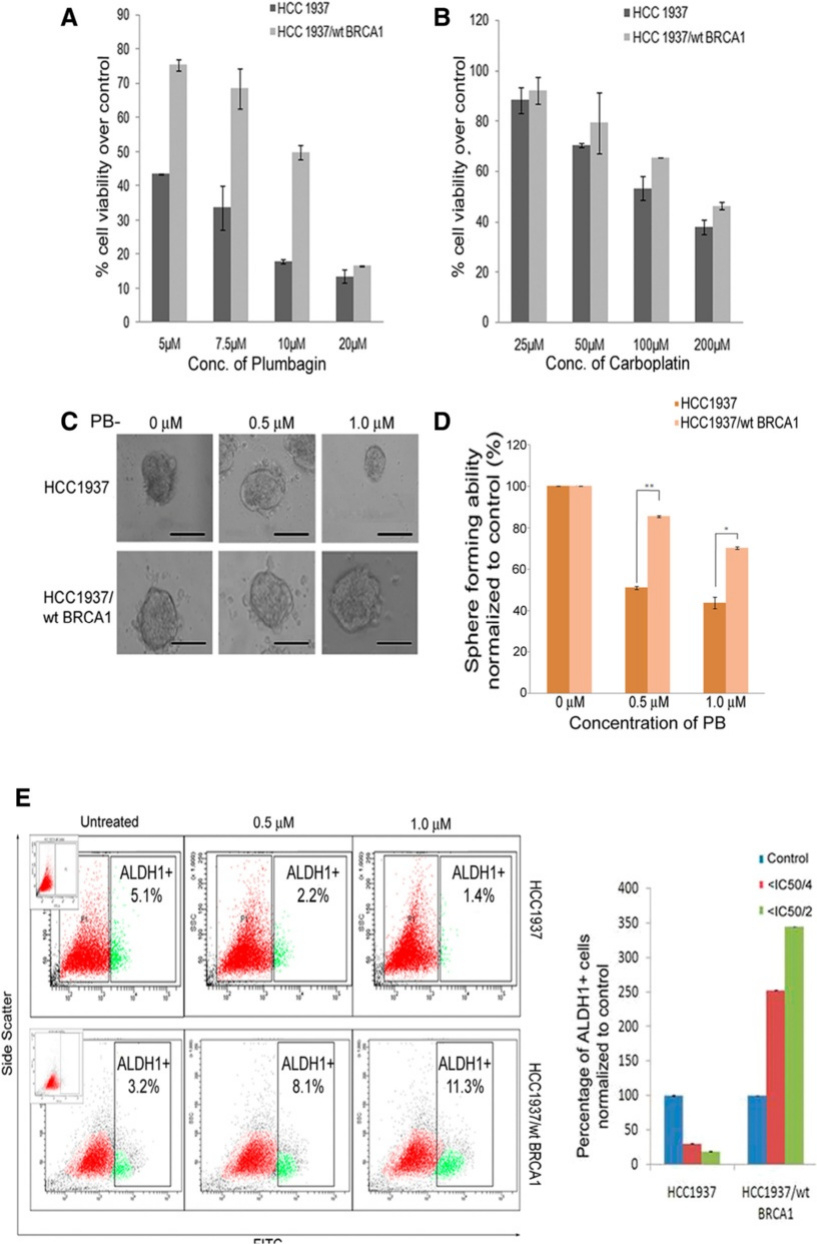
Cytotoxicity of PB is selective to BCSCs from BRCA1-defective breast cancer cell line. Effects of (**a**) Plumbagin and (**b**) Carboplatin on the proliferation of HCC1937 and HCC1937/wt BRCA1 cells. **c** PB reduces the sphere forming efficiency in terms of size and (**d**) number of spheres formed in a dose dependent manner in HCC1937 cells when compared to HCC1937/wt BRCA1 cells. Scale = 100 μm. **e** Reduction in the ALDH1+ population after PB treatment observed in HCC1937 cells while an enrichment of ALDH1+ stem cells upon PB treatment was observed in HCC1937/wt BRCA1 cells by flow cytometry and graph showing the variation in the percentages of ALDH1+ populations harbored by the breast cancer cell lines after treatment with ≤ IC_50/2_ (1 μM ) and ≤ IC_50/4_ (0.5 μM) concentrations of PB

We now went on to study if PB that has been shown to the selective to BRCA1-defective ovarian cancer cells [23] could also selectively target BRCA1-defective basal-like breast cancer cells. Immunofluorescence and western blotting were used to analyze the expression and intracellular localization of BRCA1 in HCC1937 and HCC1937/wt BRCA1 cell lines. We confirmed the low, cytosolic expression of BRCA1 in HCC1937 while in HCC1937/wt BRCA1, the BRCA1 protein was expressed and localized specifically to the nucleus (Additional file 3: Figure S2). This was expected as the truncated, non-functional BRCA1 expressed by HCC1937 has been observed in nucleus as well as the cytoplasm [32]. Cell proliferation assay revealed that PB is highly selective towards the HCC1937 cells with 5 μM concentration inhibiting proliferation in 56 % cells while in HCC1937/wt BRCA1 only 25 % inhibition of proliferation was observed (Fig. 3a, Additional file 2: Table S2). CP exhibited moderate selectivity towards BRCA1-defective cells as well, with 200 μM concentration causing 54 % growth inhibition in HCC1937/wt BRCA1 and 62 % inhibition in HCC1937 cells (Fig. 3b).

siRNA knockdown of BRCA1 showed that the HCC1937 cell line which possesses a C-terminus mutated BRCA1 did not show any difference in susceptibility to PB as assessed by MTT assay, while there was a prominent decrease in cell proliferation in the HCC1937/wt BRCA1 cells upon blocking BRCA1 (Additional file 4: Figure. S3). Thus, the activity of PB is closely linked to the BRCT domain 2 of BRCA1 that is absent in HCC1937 cell line.

### Plumbagin can target BCSCs

**Fig. 4:**
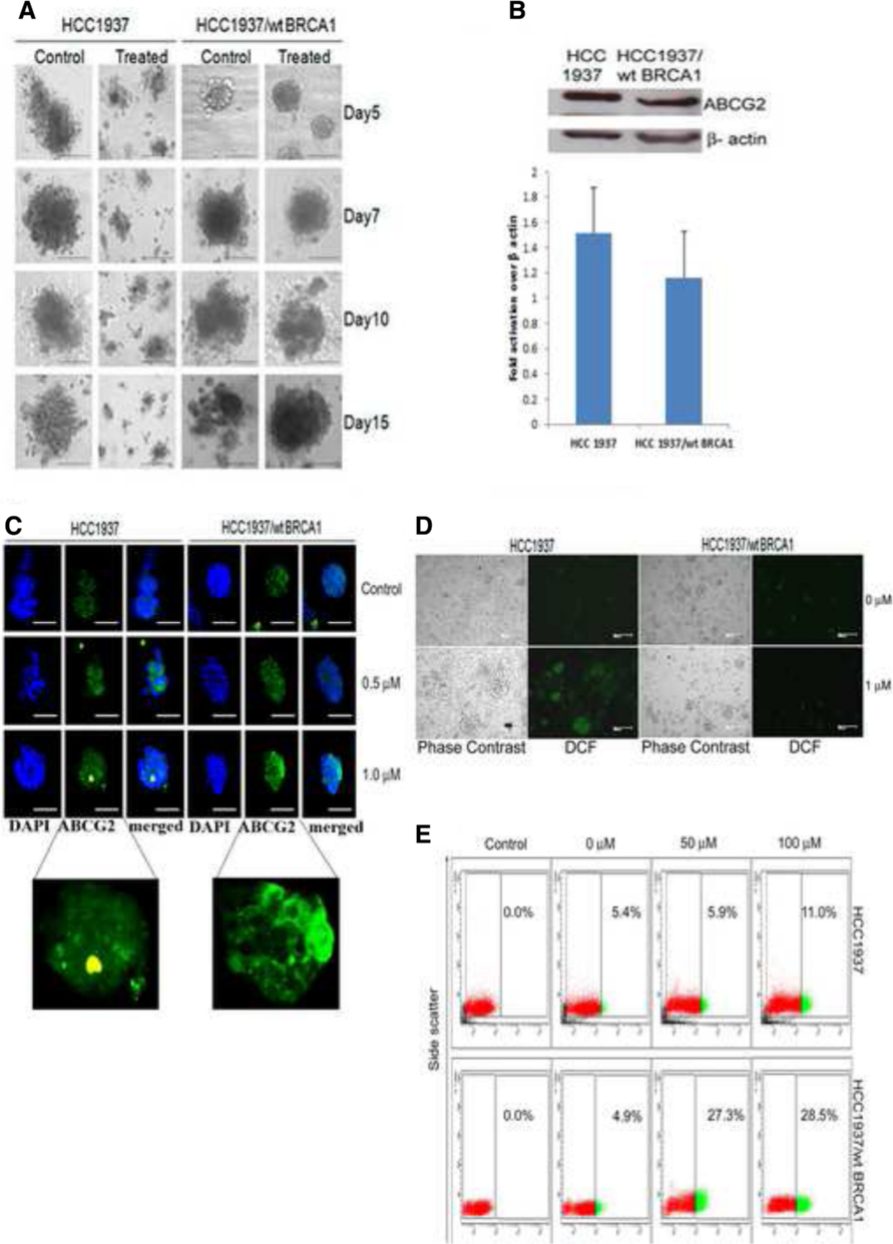
BRCA1-defect specific effects of PB are attributable to production of ROS and weak membrane localization of ABCG2. **a** 15-day Matrigel culture of mammospheres derived from HCC1937 cells and HCC1937/wt BRCA1 cells shows the selective inhibition of sphere formation in HCC1937 in the presence of 0.5 μM PB. **b** the ABCG2 expression in these cell lines. **c** ABCG2 expression in HCC1937 and HCC1937/wt BRCA1 derived mammospheres formed in presence of varying concentrations of PB shows improved membrane localization of ABCG2 after PB treatment in HCC1937/wt BRCA1 mammospheres. Cells were treated with PB on day 1, mammospheres were supplemented with growth media without PB on day 4 and mammospheres were analyzed on day 7 (**d**) Variation in the production of ROS in PB treated spheres of HCC1937 and HCC1937/ wt BRCA1 cell lines observable as a bright green fluorescence by DCF-DA staining. **e** Aldefluor assay after 48 h treatment of HCC1937 and HCC1937/wt BRCA1 cells with sub-cytotoxic concentrations of CP shows a dose dependent enrichment of ALDH1+ cells in both the cell lines. Scale = 100 μm

To assess whether the ALDH+ ‘putative CSCs’ could be specifically targeted based on the presence or absence of functional BRCA1 protein, we analyzed the effects of PB on these populations of cells. In vitro studies with regard to the BRCA1-defective scenario showed that the number of ALDH1+ cells as well as mammosphere forming efficiency significantly reduced in a dose dependent manner after treatment with < IC_50/2_ (1.0 μM) and < IC_50/4_ (0.5 μM) concentrations of PB. HCC1937 cells showed a significant dose dependent reduction in sphere forming efficiency upon PB treatment when compared to HCC1937/wt BRCA1 cells at 0.5 μM (*p* = 0.0001) and 1.0 μM (*p* = 0.01) concentrations (Fig. 3c and d). However, when the direct effect of PB on BCSC numbers was analyzed, HCC1937/wt BRCA1 cells showed a surprising enrichment of the ALDH1+ cellular compartment upon PB treatment from 3.2 % in control cells to 8.1 % in 0.5 μM and 11.3 % in 1.0 μM concentrations of PB (Fig. 3e). A dose dependent reduction in sphere formation efficiency and ALDH1+ cell numbers was indicative of the ability of PB to specifically target BRCA1-defective BCSCs. A direct inhibition of sphere formation selectively in HCC1937 in 3D culture was observed when compared to HCC1937/wt BRCA1 which showed a slight, progressive increase in size of spheres from day 5 to day 15 (Fig. 4a).

Interstingly, the graph of Aldefluor assay in Fig. 3e shows that the HCC1937/wt BRCA1 CSCs are enriched by PB treatment. This observed increase in the ALDH1+ cells within the HCC1937/wt BRCA1 cell line post PB treatment, could not be correlated directly to ABCG2 expression as has been reported earlier [33] since HCC1937 expressed higher levels of ABCG2 than HCC1937/wt BRCA1 (Fig. 4b). Immunofluorescence analysis of PB treated mammospheres showed prominent membrane localization and marginally increased expression of ABCG2 in HCC1937/wt BRCA1 cell line. However, no such change was observed in the HCC1937 derived spheroids formed in presence of PB (Fig. 4c). Hence, the increase in ALDH1+ cells in HCC1937/wt BRCA1 cells could be attributed to the more robust membrane localization rather than increased expression of the drug efflux pump ABCG2 in comparison to HCC1937.

PB was found to induce reactive oxygen species (ROS) selectively in HCC1937 mammospheres while the ROS levels of HCC1937/wt BRCA1 spheres remained unaffected as evidenced by DCF-DA staining (Fig. 4d) indicating that the oxidative stress created was effectively taken care of and hence may not be cytotoxic to HCC1937/wt BRCA1 mammospheres. We also find that DNA DSBs are more in HCC1937 than in HCC1937/wt BRCA1 when treated with PB as DNA DSBs could not be repaired probably because of the absence of functionally active BRCA1 (Additional file 5: Figure S4).

A comparison of PB with the standard drug CP was also performed. The concentrations of CP used were ≤ IC_50/2_ (100 μM) and ≤ IC_50/4_ (50 μM). When HCC1937 and HCC1937/ wt BRCA1 cells were treated for 48 h and Aldefluor assay performed, there was a dramatic increase in the number of ALDH1+ cells to 27.3 % in the 50 μM treatment and 28.5 % in the 100 μM CP treatment (Fig. 4e) from 4.0 ± 0.9 % in untreated control HCC1937/wt BRCA1 cells. An enrichment of ALDH1+ cellular sub-population to 11.0 % was observed in HCC1937 cells treated with 100 μM CP for 48 h (Fig. 4e) from 5.4 ± 0.4 % in untreated condition.

### Plumbagin can target CSCs in ovarian cancer bearing SCID mice

**Fig. 5:**
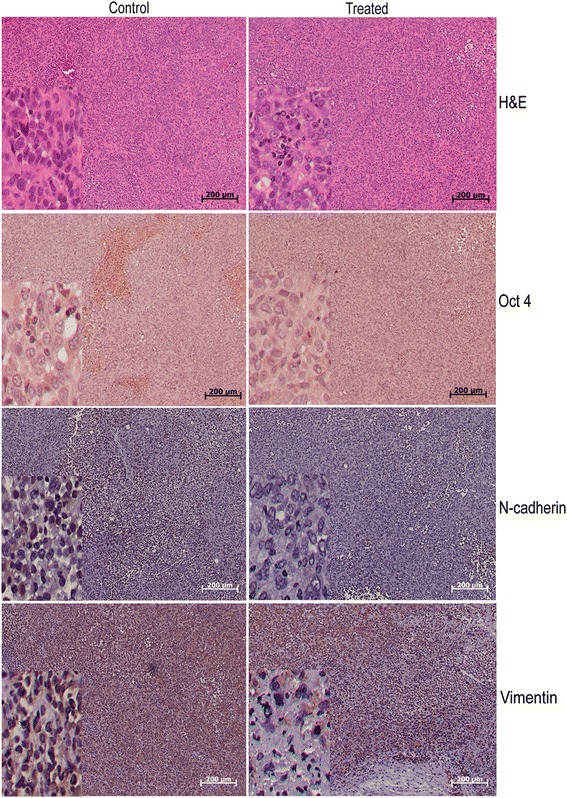
PB reduces expression of ‘stemness’ amd EMT markers in ovarian cancer xenografts. Immunohistochemical analysis of the expression of Oct 4, N-cadherin and Vimentin in OVCAR-5 xenografts before and after PB treatment. A prominent reduction in N Cad, Vimentin and Oct 4 expressing cells was observed in PB treated samples. Scale = 200 μm

To assess if the CSC targeting effects of PB could be extended to ovarian cancers as well, we performed immunohistochemical analysis on ovarian cancer xenografts to study the expression of different EMT/stem cell markers. Earlier studies from our group as well as other groups have found PB to be effective in targeting ovarian cancer cells [24]. However, plumbagin to a lesser extent targets the BRCA1- competent cells as well. To investigate this further in ovarian CSCs, OVCAR-5 xenografts in NOD/SCID mice were subjected to immunohistochemical analysis with antibodies for Oct 4, N-cadherin and Vimentin and it was found that PB treatment reduced the expression of all the three markers with the most prominent being the reduction in N-cadherin mesenchymal marker expression (Fig. 5) followed by Vimentin and Oct 4 expression. Hence, PB can putatively target ovarian CSCs by predominantly affecting the N-cadherin expressing cells and to a lesser extent by targeting the mesenchymal cells that express Vimentin and stem cells that express Oct 4. The studies on OVCAR5 xenografts provide preliminary evidence for the possibility of targeting CSCs with plumbagin.

## Discussion

Goodell et al., 1996, showed that the low Hoechst staining population could be identified as a special, drug effluxing ‘Side Population’ (SP) that conferred drug resistance to cancer cells and was responsible for relapse [34]. Subsequently, side population cells from various cancers were isolated using the property of exclusion of the Hoechst 33,342 dye and all these isolated populations showed long-term proliferative potential and the ability to differentiate into the mesenchymal cell lineage [35, 36]. The possible roles played by the drug efflux pumps in CSCs have also been elucidated with the drug resistance protein ABCB1 recently being implicated in the functioning of Renal Cell carcinoma SP cells [37]. Our results suggest the existence of SP cells with 1–2.7 % predominance across breast cancer cell lines from varying subtypes. MDA-MB-436 (1.2 ± 0.1 %) and HCC1937 (2.7 ± 0.4 %), both BRCA1-defective cell lines showed large variation in the SP abundance. No direct correlation could be drawn between BRCA1 status and SP levels as other factors governing the genotype of the cells may be important determinants. Hence, SP analysis may not be the method for identification of BRCA1-defective BCSCs across subtypes. However, a reduction in the SP cell numbers was observed when BRCA1 was introduced into HCC1937.

It was found that very small cell numbers of the CD44^+^/24^–^phenotype were capable of initiating mouse mammary tumors [6, 14]. The CD44^+^/24^–/low^ cell population also showed the properties of self renewal and multi-lineage differentiation as well and thus, this phenotype of CD44^+^/24^–^was tagged as the putative breast cancer stem cell phenotype. Studies have suggested that the percentage of CD44^+^/24^–/low^ putative CSCs present would vary between cancer cell lines and this percentage is determined by two main factors: the origin of the cell line and the receptor status [9]. When isolated from cancer tissues, the stem cell abundance is dependent on many more biological markers such as tumor size, histological grade, lymph node lesions, receptor status and molecular subtype of the tumor [33, 38, 39]. A comparison of the sub-cellular percentages of these populations in five different cell lines brought to light the fact that the molecular subtype of the tumor induced by the cell lines and the BRCA1 status were important in determining the CD44^+^/24^–/low^ and the ALDH1+ cell population numbers respectively. This is in addition to the already known determinant namely, the cell type of origin [9]. It was observed that the BRCA1- mutated cell lines were enriched for ALDH1+ cells in both normal-like tumor forming (MDA-MB-436) and basal-like tumor forming (HCC1937) cell lines. ALDH1 positivity was thus associated with a BRCA1 defect as reported earlier [20] and we additionally find this association to be independent of tumor sub-type unlike the CD44/24 profile. A similar link was also observed between the CD44^+^/24^–/low^ profile and BRCA1 expression status in basal–like breast cancer cells, as HCC1937 expressed higher CD44^+^/24^–/low^ cells than the HCC1937/wt BRCA1 cell line. Estrogen Receptor and Progesterone Receptor positive MCF7 cell line exhibited the least number of SP, CD44^+^/24^–/low^ and ALDH1+ cells. This could be due to its low invasive capabilities in comparison to the TNBC cell lines [40]. The aggressive TNBCs of basal origin showed a high expression of CD44^+^/24^–/low^ cells with MDA-MB-231 and MDA-MB-436 that formed normal like breast cancers exhibiting higher levels of these putative BCSCs. The existence of 80-90 % CD44^+^/24^–/low^ cells in MDA-MB-231 and MDA-MB-436 adds credence to earlier reports that this population is not exclusively constituted of BCSCs [41]. Further, the number of CD44^+^/24^–/low^ cells was significantly lower in HCC1937 when compared to MDA-MB-436 (*p* = 0.0001) though both were BRCA1-mutated. Thus, the CD44^+^/24^–/low^ population abundance could be independent of BRCA1 expression and dependent on the tumor sub-type.

The use of the three established methods for isolation of CSCs followed by in vitro self renewal analysis by mammosphere formation studies have thus brought out the ALDH1+ phenotype as the best candidate putative stem cell marker for BRCA1-defective breast cancer cells. This is because the ALDH1+ cells from BRCA1-defective cell lines formed large mammospheres in comparison to ALDH1+ cells from BRCA1-competent breast cancer cells. Also, the ALDH1- cells from HCC1937 and MDA-MB-436 cells failed to form spheroids in culture. The ALDH1- cells from MCF7 and HCC1937/ wt BRCA1 possessing a wild type BRCA1 gene formed spheroids though fewer and smaller in size than those formed by the corresponding ALDH1+ cells.

A recent report indicates ALDH1 positivity to be a better marker for relapse than the CD44^+^/24^–/low^ profile [42]. Also, the CD44^+^/24^–/low^ profile has been found to represent the slow cycling, mesenchymal BCSCs while the ALDH1+ cells are the rapidly proliferating epithelial BCSCs [43]. This being the case, we decided to analyze the HCC1937/wt BRCA1 and HCC1937 cells for their mesenchymal and proliferative characteristics. Immunofluorescence based profiling of the spheroids generated from the two cell lines indeed showed a more robust expression of the EMT markers Snail, Slug and Vimentin and mesenchymal marker α-SMA in the HCC1937/wt BRCA1 mammospheres and a higher expression of the stem cell markers Oct 4 and β-catenin in the HCC1937 cell line. Concordantly, we also observed for the first time the existence of a CD44^high^/24^–^population within the HCC1937/wt BRCA1 cell line which is absent in HCC1937 cells, possibly responsible for the prominent mesenchymal nature of HCC1937/wt BRCA1 cells that showed the least in vitro sphere forming ability while the HCC1937 harboring proliferating stem cells formed the maximum number of spheres in culture as shown in Fig. 1d.

Earlier reports from our group have established the ability of PB, a naturally occurring naphthoquinone to induce cytotoxicity specifically and selectively in BRCA1 defective ovarian cancers [21–24, 44]. The C-terminal end of the BRCA1 protein has been shown to be involved in phospho-protein binding thus playing a direct, central role in the functioning of the protein [45, 46]. HCC1937 has a 5382insC mutation that leads to the synthesis of a C-terminal truncated BRCA1 protein [47, 48]. The silencing of BRCA1 and associated difference in susceptibility of HCC1937/wt BRCA1 to PB show that a mutated BRCT domain 2 at the C-terminus of BRCA1 plays an important role in mediating the specific activity of PB in BRCA1-defective cancers. Hence, we suggest that the activity of PB is closely linked to the presence of a functional BRCT domain 2 of BRCA1.

**Fig. 6:**
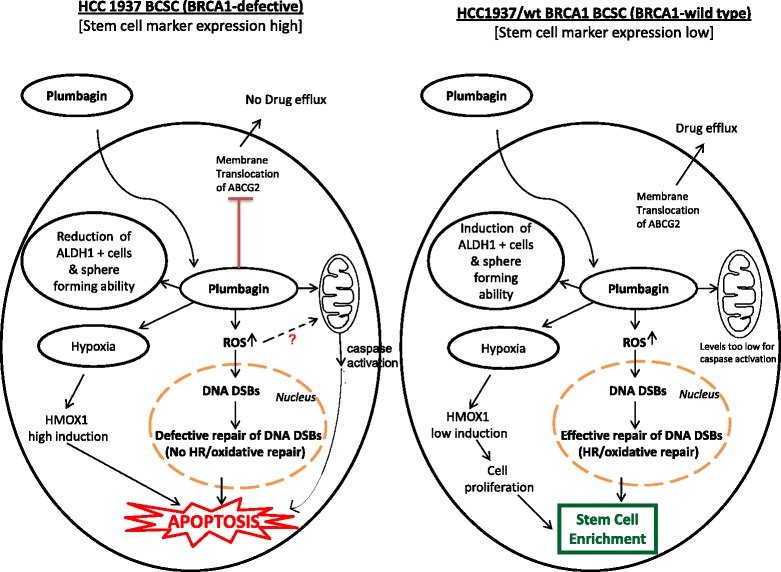
Model of BRCA1 defective BCSCs and response to anticancer agents. BRCA1 defective BCSCs are mostly positive for stem cell markers (Nuclear Oct 4 and membrane bound β-catenin) while BRCA1 wild type BCSCs shows expression of EMT markers (Snail, Slug, Vimentin) and mesenchymal marker (α-SMA). PB, an ROS inducer can cause DNA DSBs which cannot be effectively repaired in BRCA1 defective cells leading to apoptosis (unpublished data). Also, very high levels of HMOX1 expression in BRCA1 deficient condition as reported earlier, may cause apoptosis induction. ROS mediated DNA damages will be repaired and low levels of hypoxia created by PB can lead to low HMOX1 induction and further stem cell enrichment in BRCA1 wild type BCSCs. In addition to this, drug efflux will not happen as ABCG2 membrane translocation is hampered in presence of PB in BRCA1 defective mammospheres. Due to the presence of active ABCG2, drug efflux will also be high when treated with PB in BRCA1-wild type BCSCs. The same mechanism may be in action in both the conditions after CP treatment

A number of varied targets of plumbagin have been identified over the years [26] that effected cell viability by the induction of G2-M arrest, autophagy, apoptosis as well as inactivation of NK-kB and Bcl2 [27, 49]. However, the possible effects of PB in CSCs are still unexplored and have been analyzed in this study. An agent that has a putative ability to target CSCs exhibits a three-pronged effect in vitro by inducing a reduction in number and size of mammospheres as well as directly reducing or abrogating the CSC marker expressing population. Such a decrease in ALDH1+ cells associated with Wnt signaling was observed by Y Li et al., 2010 with sulphorafane treatment in breast cancer cells [50]. In vitro experiments to study the effects of PB on BCSCs gave promising activity where as the standard drug carboplatin caused an increase in the ALDH1+ stem cell numbers post treatment. This observed increase after treatment is concordant with carboplatin being an effective anti-cancer drug capable of killing and clearing off the bulk tumor cells rapidly. This consequently triggers the otherwise quiescent stem cells (ALDH1+ cells) to enter the cell cycle, thus leading to a dramatic increase in the number of ALDH1+ ‘stem cells’ and possibly accounting for the few incidences of relapse after chemotherapy with carboplatin. This enrichment of ALDH1+ BCSCs in PB treated HCC1937/wt BRCA1 could be explained as a direct effect of membrane localization rather than increased expression of the drug efflux pump ABCG2 (Fig. 4e). However, in HCC1937, PB shows the promising ability to directly reduce the number of ALDH1+ BCSCs possibly by inhibiting the membrane localization of ABCG2, as well as by triggering ROS generation [51, 52]. The ROS signaling induced by PB could be through the activation of PI5K-1B protein that then induces ROS production and/ or triggers loss of mitochondrial membrane potential, and apoptosis [23, 53]. This further corroborates the specificity of PB to target BRCA1-defective cells and also provides a reason for the BCSC enrichment observed in PB treated HCC1937/wt BRCA1 spheres in which the ROS induction by PB is seen to be effectively nullified and hypoxic condition is maintained. Hypoxia is a well-documented inducer of EMT as well as CSC phenotypes [54–56]. We have shown earlier that HMOX1 is about 6.6 fold high in BRCA1 deficient than BRCA1 proficient condition when treated with plumbagin [44]. The sustained high levels of ROS and corresponding high levels of HMOX1, compared to moderate-low levels of ROS and HMOX1 observed in BRCA1 wild type condition, could induce apoptosis in BRCA1-deficient condition instead of survival. Thus, PB which is reported earlier to have a specific cytotoxicity in BRCA1-defective ovarian cancers, is found in this study to possess the ability to target BRCA1-defective BCSCs as well (Fig. 6). Recent studies have found the involvement of the Wnt/β-catenin-ABCG2 signaling pathway in chemoresistance and tumor-initiating capacity of ovarian cancer cells [57]. However, none of these have studied the differences in sub-cellular localization of ABCG2.

Immunohistochemical analysis of ovarian cancer xenografts has provided preliminary in vivo evidence for the ability of PB to target CSCs. A reduction in Vimentin (EMT marker), N-cadherin (Mesenchymal marker) as well as Oct 4 (Stem cell marker) expressing cells after PB treatment shows for the first time, the ability of the naphthoquinone to target CSCs in vivo. Studies from our group have earlier shown that Plumbagin can target BRCA1-defective breast (unpublished data) as well as ovarian cancers [21] more effectively than it can target BRCA1-competent cells from both cancers. The studies on OVCAR5 xenografts provide preliminary evidence for the possibility of targeting BCSCs with plumbagin. This could be accomplished with greater selectivity to BRCA1-defective BCSCs if the in vitro effects of PB are reflected in vivo too. This paves way for further xenograft experiments with BRCA1-defective breast cancer cell lines which could bring out the specific and pronounced effects of PB in BRCA1-defective BCSCs as is expected from the in vitro and in vivo data generated from this study.

This study shows a possible role for BRCA1 in determining the EMT and stem cell characteristics of BCSCs and also links BRCA1 and the marker expression profile of BCSCs to the existence of a CD44^high^/24^–^sub-population. In support of this, suppression of EMT by BRCA1 and expression of Slug and Snail in response to repression of BRCA1 expression has been reported earlier [58, 59].

It has been very well proved that low levels of ROS help CSCs to survive and lead to adaptive changes for tumor progression. Shi et al., 2012 demonstrated that, the ROS generated by xenobiotics tip the redox balance and kill cancer cells while not affecting normal cells [60]. In our study, the ability of PB to induce DSBs (Additional file 5: Figure S4) coupled with its ability to generate ROS in HCC1937 mammospheres (Fig. 4d) leads to a ‘double effect’ where the increased ROS in the mammospheres stresses the CSCs that are known to thrive in low ROS conditions and the additional burden of DNA DSBs that cannot be repaired due to a lack of functional BRCA1 causes the CSCs to die. This is reflected in the reduction of ALDH1+ BCSC population after PB treatment. On the other hand, PB is unable to induce ROS, DNA DSBs in HCC1937/wt BRCA1 BCSCs which continue to thrive in the lower ROS environments present in the PB treated mammospheres. This leads to the increase in ALDH1+ BCSC numbers after PB treatment. This is the maiden study addressing the ability of PB to target CSCs with selectivity for BCSCs from BRCA1-defective breast cancer cell lines. At the molecular level, ROS induced by PB in the BCSCs may be working partially through the Wnt/β catenin-ABCG2 pathway to make BCSCs more susceptible to PB by preventing its efflux [57, 61]. This aspect of the action of PB has to be investigated further.

Thus, future studies aimed at targeted delivery of PB into BRCA1-defective breast cancers could open up new avenues for the complete abrogation of the cancer, including CSCs, aiding relapse-free survival after chemotherapy.

## Conclusion

In support of the recalcitrant nature of BRCA1-related cancers to therapy, we show the predominant expression of stem cell markers in mammospheres derived from BRCA1-defective cancer cells in contrast to the EMT markers expressed by BCSCs in a BRCA1-competent condition. Majority of chemotherapeutics abrogate rapidly proliferating bulk tumor cells, while quiescent, therapy resistant CSCs are subsequently enriched. We demonstrate for the first time that a naphthoquinone, PB selectively reduces the ALDH1+ population in basal-like BRCA1-defective breast cancer cells while enriching the ALDH1+ population in cancer cells harboring full length, functional BRCA1, the reason for which has to be analyzed in future. Carboplatin on the other hand, caused an enrichment of ALDH1+ cells irrespective of BRCA1 status. PB also induces ROS production- potentially culminating in cell death- exclusively in the mammosphere-derived cells of BRCA1-defective HCC1937. This study gives insights for an effective treatment regimen that can inhibit CSC-induced resistance to therapy and subsequent disease relapse.

## Abbreviations

ALDH1, Aldehyde dehydrogenase 1; BCSCs, Breast cancer stem cells; CP, Carboplatin; CSCs, cancer stem cells; DCF-DA, Dichloro-dihydro-fluorescein diacetate; DSB(s), Double strand break(s); EMT, Epithelial-mesenchymal transition; HIF, Hypoxia inducible factors; HMOX1, heme oxygenase-1; HR, Homologous recombination; MET, Mesenchymal-epithelial transition ; PB, Plumbagin; ROS, Reactive oxygen species; SP, side population; TNBC, triple negative breast cancer.

## Additional files

Additional file 1: Figure S1. Contour plot showing the distinct CD44high/24- population (indicated by black arrow) in HCC1937/wt BRCA1 cell line in support of Fig. 2a. Here, the existence of the CD44high/24^-/low^ cell sub population possessing mesenchymal properties in HCC1937/wt BRCA1 is more evident. (B) Expression and localization of BRCA1 in HCC1937 and HCC1937/wt BRCA1 cell lines. (TIF 27522 kb)

Additional file 2: Table S1 and S2. **Table S1. ** The fold change values generated using the GeneSpring software after Microarray analysis of the HCC1937 mammospheres in comparison to the HCC1937/wt BRCA1 mammospheres and are indicative of the genes whose expression is directly or indirectly linked to BRCA1. **Table S2.** IC_50_ values of Plumbagin (PB) and Carboplatin (CP). (DOCX 11 kb)

Additional file 3: Figure S2. Expression and localization of BRCA1 in HCC1937 and HCC1937/wt BRCA1 cell lines by immunofluorescence and western blotting. Quantitation of BRCA1 expression normalized with β-actin in western blot by densitometry analysis is also indicated. (TIF 28769 kb)

Additional file 4: Figure S3. Cytotoxic effect (by MTT assay) of PB (24 h treatment) in HCC1937 (A) and HCC1937/wt BRCA1 (B) after the siRNA mediated blocking of BRCA1. HCC1937 and HCC1937/wt BRCA1 cells were treated for 48 h with full length 2.4pM siRNA for BRCA1 (Eurogentec, Liège, Belgium) (siRNA Sense (+dTdT), 19 bases in length, BRCA1 position 1857–1879, GGUCAAGUGAUGAAUAUUA) as per manufacturer’s instructions followed by treatment with PB for 24 h. (TIF 9107 kb)

Additional file 5: Figure S4. Comet Assay: DSB induced by PB (8 h treatment) in HCC1937 (A) and HCC1937/wt BRCA1 (B) observed by comet assay. HCC1937 and HCC1937/wtBRCA1 cells were treated for 8 h with varying concentrations of PB (2.5uM, 5uM and 7uM) and comet assay performed after neutral lysis of the treated cells. The top two panels show the HCC1937 and HCCC1937/wt BRCA1 cells. Tails of damaged DNA are visible in HCC 1937 cells after 5 uM and 7uM treatment with PB. The graph quantifies the Olive tail moment indicating the extent of DNA damage. (TIF 2882 kb)

Additional file 6: Supplementary materials and methods. (DOCX 13 kb)
