# Supplementary figures and images for: Selective mode of action of plumbagin through BRCA1 deficient breast cancer stem cells
Source: BMC Cancer. 2016 May 26;16:336. doi: 10.1186/s12885-016-2372-4 (PMC4882782; doi:10.1186/s12885-016-2372-4)

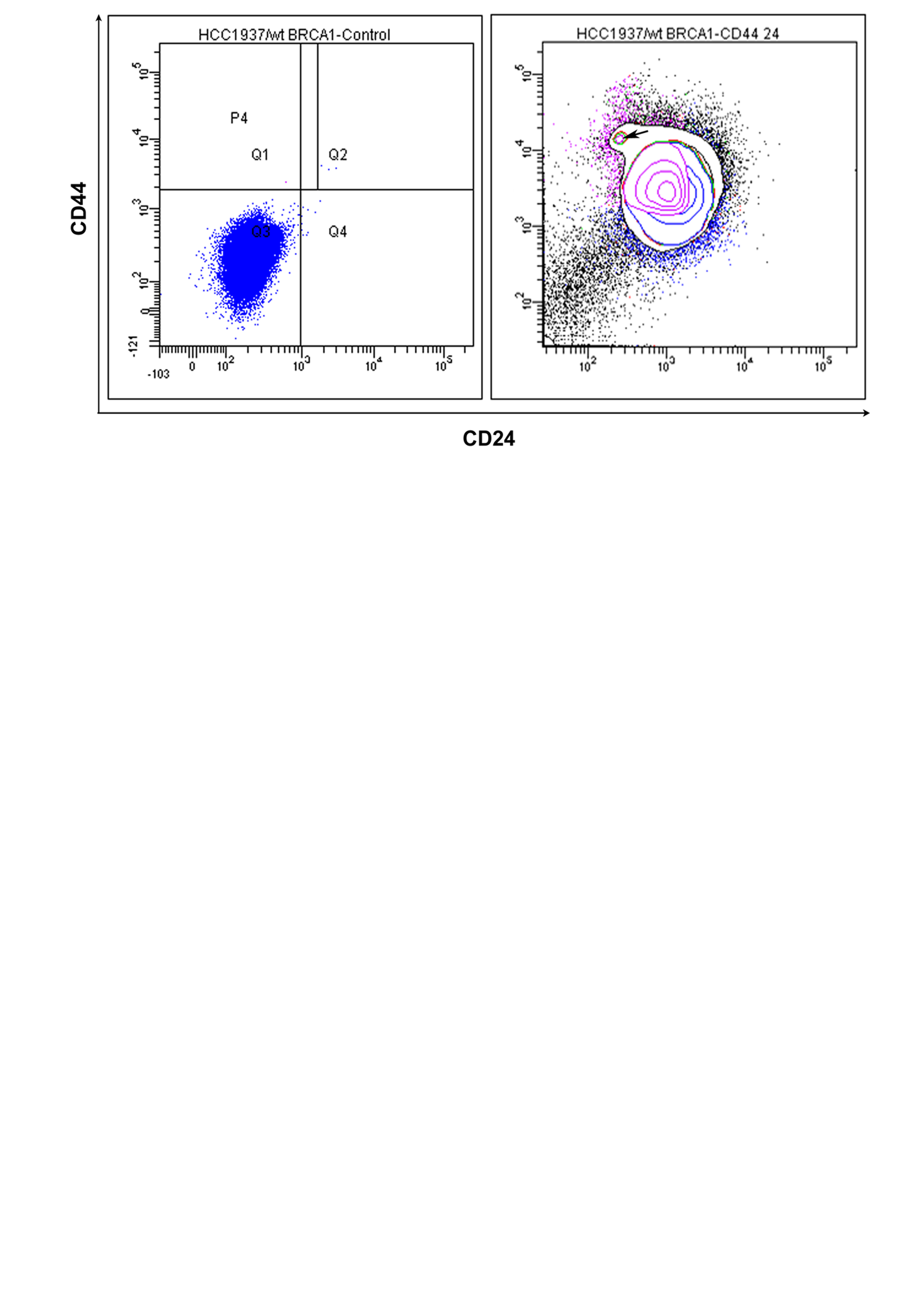

Supplement: Supplementary file 1 — Contour plot showing the distinct CD44high/24- population (indicated by black arrow) in HCC1937/wt BRCA1 cell line in support of Fig. 2a. Here, the existence of the CD44high/24-/low cell sub population possessing mesenchymal properties in HCC1937/wt BRCA1 is more evident. (B) Expression and localization of BRCA1 in HCC1937 and HCC1937/wt BRCA1 cell lines. (TIF 27522 kb) [file 12885_2016_2372_MOESM1_ESM.tif]

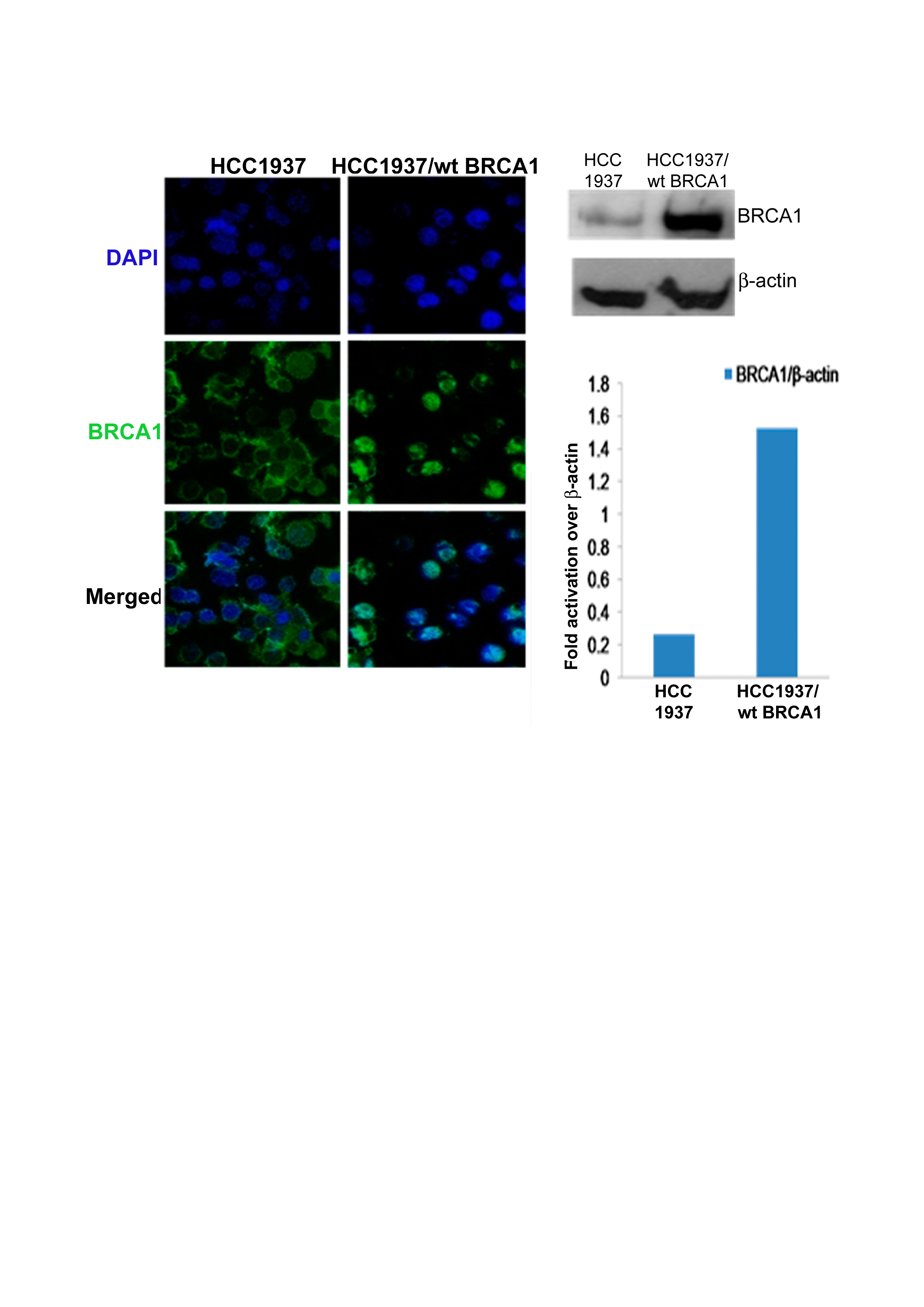

Supplement: Supplementary file 3 — Expression and localization of BRCA1 in HCC1937 and HCC1937/wt BRCA1 cell lines by immunofluorescence and western blotting. Quantitation of BRCA1 expression normalized with β-actin in western blot by densitometry analysis is also indicated. (TIF 28769 kb) [file 12885_2016_2372_MOESM3_ESM.tif]

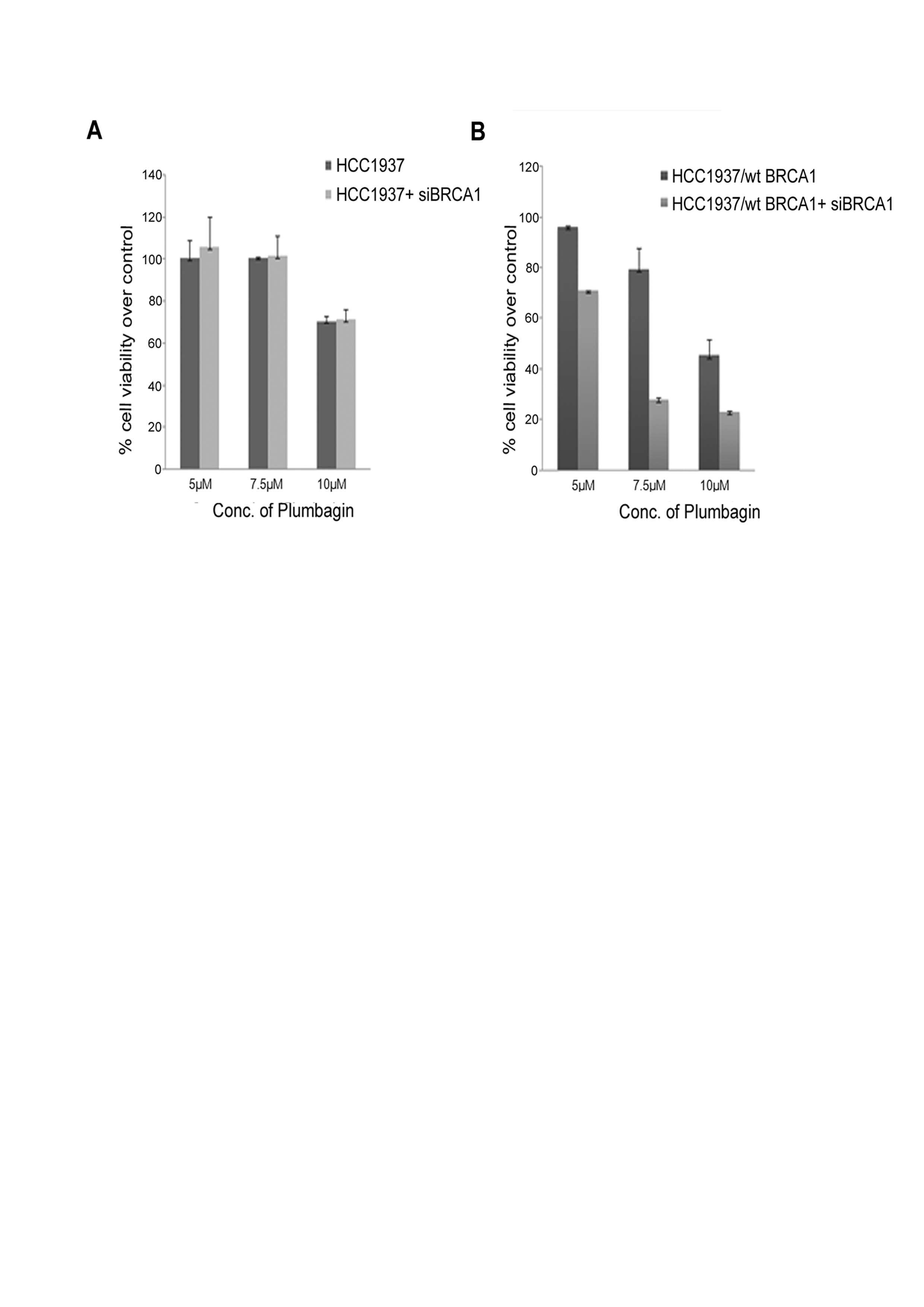

Supplement: Supplementary file 4 — Cytotoxic effect (by MTT assay) of PB (24 h treatment) in HCC1937 (A) and HCC1937/wt BRCA1 (B) after the siRNA mediated blocking of BRCA1. HCC1937 and HCC1937/wt BRCA1 cells were treated for 48 h with full length 2.4pM siRNA for BRCA1 (Eurogentec, Liège, Belgium) (siRNA Sense (+dTdT), 19 bases in length, BRCA1 position 1857–1879, GGUCAAGUGAUGAAUAUUA) as per manufacturer’s instructions followed by treatment with PB for 24 h. (TIF 9107 kb) [file 12885_2016_2372_MOESM4_ESM.tif]

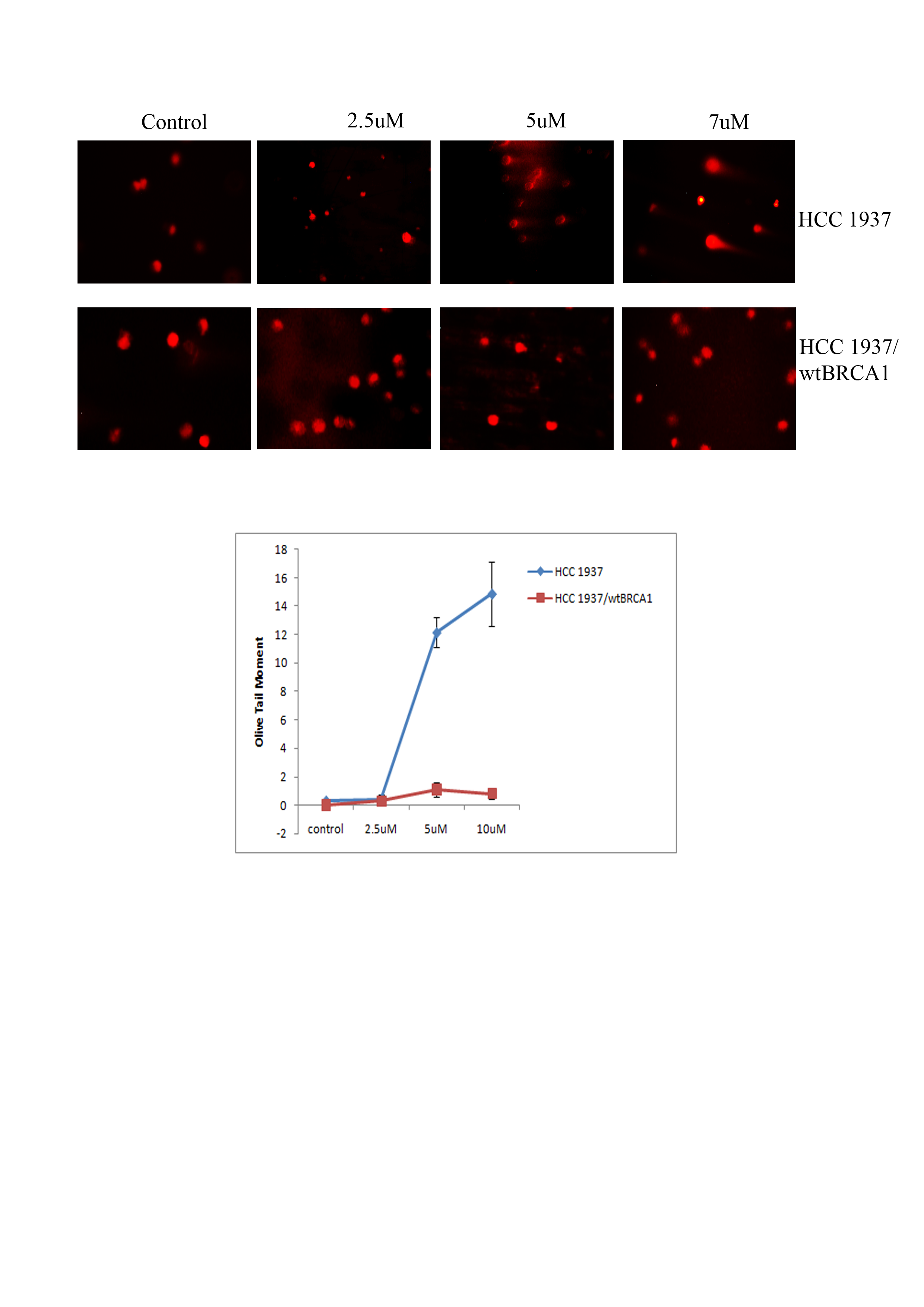

Supplement: Supplementary file 5 — Comet Assay: DSB induced by PB (8 h treatment) in HCC1937 (A) and HCC1937/wt BRCA1 (B) observed by comet assay. HCC1937 and HCC1937/wtBRCA1 cells were treated for 8 h with varying concentrations of PB (2.5uM, 5uM and 7uM) and comet assay performed after neutral lysis of the treated cells. The top two panels show the HCC1937 and HCCC1937/wt BRCA1 cells. Tails of damaged DNA are visible in HCC 1937 cells after 5 uM and 7uM treatment with PB. The graph quantifies the Olive tail moment indicating the extent of DNA damage. (TIF 2882 kb) [file 12885_2016_2372_MOESM5_ESM.tif]
