# Supplementary material for: Selective mode of action of plumbagin through BRCA1 deficient breast cancer stem cells
Source: BMC Cancer. 2016 May 26;16:336. doi: 10.1186/s12885-016-2372-4 (PMC4882782; doi:10.1186/s12885-016-2372-4)
Supplement: Supplementary file 2 — Table S1. The fold change values generated using the GeneSpring software after Microarray analysis of the HCC1937 mammospheres in comparison to the HCC1937/wt BRCA1 mammospheres and are indicative of the genes whose expression is directly or indirectly linked to BRCA1. Table S2. IC50 values of Plumbagin (PB) and Carboplatin (CP). (DOCX 11 kb) [file 12885_2016_2372_MOESM2_ESM.docx]

| **Table S1** | | |
| --- | --- | --- |
| **Downregulation of EMT markers in HCC1937 mammospheres compared to HCC1937/wt BRCA1 mammospheres** | | |
| **Gene** | **GeneBank ID** | **Fold Activation** |
| SNAI1, Snail homolog 1 | NM_005985 | 0.171 |
| SNAI2, Snail Homolog 2 | NM_003068 | -6.838 |
| SNAI2, Slug mRNA | U97060 | -2.807 |
| TWIST1 | NM_000474 | -2.171 |

| Table S2 | | |
| --- | --- | --- |
|  | **Plumbagin** | **Carboplatin** |
| **HCC1937** | 10.45μM | 173.5μM |
| **HCC1937/wtBRCA1** | 4.93μM | 140.4μM |
