# Supplementary material for: Selective mode of action of plumbagin through BRCA1 deficient breast cancer stem cells
Source: BMC Cancer. 2016 May 26;16:336. doi: 10.1186/s12885-016-2372-4 (PMC4882782; doi:10.1186/s12885-016-2372-4)
Supplement: Supplementary file 6 — Supplementary materials and methods. (DOCX 13 kb) [file 12885_2016_2372_MOESM6_ESM.docx]

**Supplementary Materials and Methods**

1. **siRNA transfection.** HCC1937 and HCC1937/wt BRCA1 cells were treated for 48 h with full length 2.4pM siRNA for BRCA1 (Eurogentec, Liège, Belgium) (siRNA Sense (+dTdT), 19 bases in length, BRCA1 position 1857-1879, GGUCAAGUGAUGAAUAUUA) as per manufacturer’s instructions followed by treatment with PB for 24 h. Effects on cell proliferation were analyzed by MTT assay as described elsewhere (21).
